# Distinct TLR- and NLR-Mediated Transcriptional Responses to an Intracellular Pathogen

**DOI:** 10.1371/journal.ppat.0040006

**Published:** 2008-01-11

**Authors:** Jess H Leber, Gregory T Crimmins, Sridharan Raghavan, Nicole P Meyer-Morse, Jeffery S Cox, Daniel A Portnoy

**Affiliations:** 1 Department of Molecular and Cell Biology, University of California, Berkeley, California, United States of America; 2 Department of Microbiology and Immunology, University of California, San Francisco, California, United States of America; 3 School of Public Health, University of California, Berkeley, California, United States of America; Yale University School of Medicine, United States of America

## Abstract

How the innate immune system tailors specific responses to diverse microbial infections is not well understood. Cells use a limited number of host receptors and signaling pathways to both discriminate among extracellular and intracellular microbes, and also to generate responses commensurate to each threat. Here, we have addressed these questions by using DNA microarrays to monitor the macrophage transcriptional response to the intracellular bacterial pathogen Listeria monocytogenes. By utilizing combinations of host and bacterial mutants, we have defined the host transcriptional responses to vacuolar and cytosolic bacteria. These compartment-specific host responses induced significantly different sets of target genes, despite activating similar transcription factors. Vacuolar signaling was entirely *MyD88*-dependent, and induced the transcription of pro-inflammatory cytokines. The *IRF3*-dependent cytosolic response induced a distinct set of target genes, including *IFNβ*. Many of these cytosolic response genes were induced by secreted cytokines, so we further identified those host genes induced independent of secondary signaling. The host response to cytosolic bacteria was reconstituted by the cytosolic delivery of L. monocytogenes genomic DNA, but we observed an amplification of this response by *NOD2* signaling in response to MDP. Correspondingly, the induction of *IFNβ* was reduced in *nod2* ^−/−^ macrophages during infection with either L. monocytogenes or Mycobacterium tuberculosis. Combinatorial control of *IFNβ* induction by recognition of both DNA and MDP may highlight a mechanism by which the innate immune system integrates the responses to multiple ligands presented in the cytosol by intracellular pathogens.

## Author Summary

Macrophages are critical cells of the innate immune system, contributing to immediate and robust defense against microbial infections. Macrophages detect pathogens using host receptors located on the cell surface, in phagosomal vacuoles, and in the cytosol. While fundamental to innate immunity, it is not clear if these different receptors merely provide redundant mechanisms for sensing microbial infection, or if instead they induce distinct gene expression programs that may allow for threat-specific host responses. We addressed this question by dissecting the macrophage transcriptional responses to the model intracellular bacterial pathogen Listeria monocytogenes. Using genetic and genomic approaches, we found that the macrophage response to L. monocytogenes trapped in phagosomal compartments was distinct and separable from the response to live bacteria replicating in the host cytosol. The macrophage response to cytosolic bacteria was recapitulated by bacterial nucleic acid and cell wall fragments, and induced surprisingly few primary response genes. These findings highlight a mechanism by which the innate immune system may specifically sense intracellular bacteria, as the macrophage response to Mycobacterium tuberculosis was similarly regulated.

## Introduction

As sentinels of the immune system, macrophages must be able to determine the nature and scope of microbial threats to mount appropriate transcriptional responses [1,2]. Macrophages need to discriminate not only viral from bacterial infection, but also extracellular and possibly killed microbes from intracellular and replicating pathogens [3]. Cells receive information regarding infection using a limited number of pattern recognition receptors (PRRs) to sense conserved motifs presented by microbes [1,4–6]. Two major classes of PRRs include membrane-bound Toll-like receptors (TLRs) and soluble, cytosolic NOD-like receptors (NLRs). TLRs monitor the extracellular environment and phagolysosomal compartments, and recognize pathogen associated molecular patterns (PAMPs) that include lipopolysaccharide, flagella, CpG DNA, and bacterial lipoprotein [7]. NLRs complement this host defense by providing surveillance of the cytosol. The nucleotide-binding and oligomerization domain containing (NOD) proteins, for which this class of receptors is named, recognize cell wall fragments from both Gram-negative and Gram-positive bacteria [8–10]. NOD1 recognizes a specific peptidoglycan (PGN) fragment containing diaminopimelic acid, while NOD2 recognizes a muramyl dipeptide (MDP) fragment of PGN. The MDA5 and RIG-I NLRs detect cytosolic dsRNA [11], while DAI detects cytosolic dsDNA [12–14].

How cells initiate a threat-specific transcriptional response is poorly understood, as many PRRs, in response to different stimuli, utilize the MyD88 and TRIF signaling adaptor molecules to activate the same transcription factors [15]. For instance, activation of both TLRs and NLRs causes degradation of the repressor IκB, thereby freeing the transcription factor NFκB to enter the nucleus and bind to target promoters of genes important for host defense. Some target specificity is generated by the phosphorylation and activation of the IRF3 transcription factor by only a subset of PRRs, including those that recognize nucleic acids. The induction of certain host genes, including the Type I interferons α (*IFNα*) and β (*IFNβ*), requires both NFκB and IRF3 [16–18]. Secreted Type I interferons then induce many additional genes by secondary signaling through the Type I interferon receptor (*IFNAR*) [15].

L. monocytogenes is a ubiquitous Gram-positive intracellular bacterium that can cause serious illness in pregnant women and immunocompromised individuals [19], and is ideal for the study of host innate immune responses. Mutants defective in precise stages of the intracellular life cycle have been isolated, and *in vitro* infection of primary bone marrow–derived macrophages allows dissection of host signaling pathways. Approximately 30 minutes after initial phagocytic uptake into a membrane-bound vacuole, bacteria escape to the host cytosol by perforating the vacuolar membrane, using the pore-forming toxin listeriolysin O (LLO, encoded by the gene *hly*) [20]. Once in the host cytosol, L. monocytogenes replicates robustly, and uses a system of actin-based motility to spread from the initially infected macrophage to colonize neighboring cells [21]. Both heat-killed and *hly− L. monocytogenes* induce inflammatory cytokines, but in non-activated macrophages only wild-type (WT) bacteria that are able to access the host cytosol induce Type I interferons [22–24].

Although the L. monocytogenes Type I interferon-stimulating ligand has not been conclusively identified, evidence suggests that bacterial DNA possesses *IFNβ*-inducing activity and might be the relevant PAMP [12,25]. PGN fragments have also been shown to induce host transcriptional responses [3,8,26–28], and MDP is present both in digested L. monocytogenes PGN fragments [29,30] and in polymer-linked form in intact PGN [31]. The role of MDP during the host response to L. monocytogenes is not clear, however [3,24,32].

In this study, we have comprehensively determined the macrophage transcriptional responses to L. monocytogenes using DNA microarrays. Using macrophages deficient for defined host signaling pathways, and bacteria residing in different subcellular localizations, we have delineated distinct host responses to vacuolar and cytosolic bacteria, and addressed the mechanisms underlying the specificity of these responses. We have additionally determined the direct transcriptional targets of host NLR signaling in response to cytosolic bacteria. These genes are co-regulated with *IFNβ*, and are uniquely induced in infected cells, as their induction is independent of any secondary signaling. These primary targets have the potential to modify host signaling, and may therefore critically impact the host response to infection. Cytosolic delivery of purified bacterial genomic DNA reconstituted the host response to cytosolic L. monocytogenes. This response was synergistically amplified by *NOD2* signaling in response to MDP. We find a similar role for *NOD2* signaling in the host response to both L. monocytogenes and M. tuberculosis, and this may represent a mechanism by which cells integrate multiple PRR signals to accurately identify bacteria able to access the host cytosol.

## Results

### Identification of Distinct Macrophage Transcriptional Responses to Vacuolar and Cytosolic L. monocytogenes

**Figure 1:**
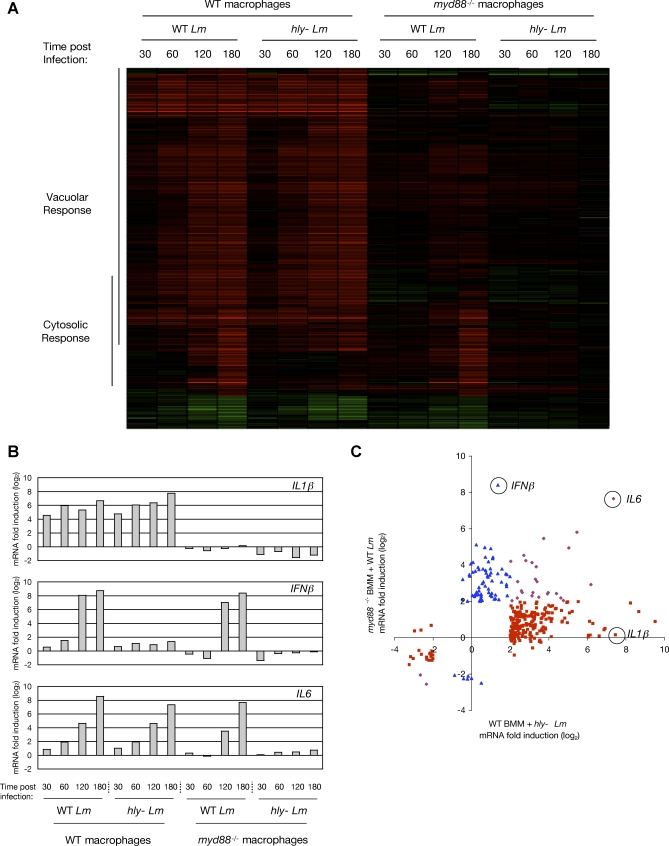
Macrophages Have Distinct Transcriptional Responses to Vacuolar and Cytosolic L. monocytogenes Infection (A) Cluster analysis of the microarray determination of all mouse macrophage genes with at least a 4-fold change in abundance during infection of WT and *myd88^−/−^* macrophages with either WT or *hly− L. monocytogenes*, at the indicated times post-infection (in minutes). Red indicates an increase in RNA abundance relative to uninfected macrophages, and green indicates a decrease. Genes identified by SAM and at least 4-fold induced in WT macrophages by *hly− L. monocytogenes* are indicated as targets of the “Vacuolar Response”. Genes identified by SAM and at least 4-fold induced in *myd88* ^−/−^ macrophages by WT L. monocytogenes are indicated as targets of the “Cytosolic Response”. For target gene lists, see Datasets S1 and S2. The determination of Vacuolar Response and Cytosolic Response target genes was from multiple arrays representing four independent experiments (*e.g.* four independent dishes of uninfected *myd88* ^−/−^ macrophages and four independent dishes of *myd88* ^−/−^ macrophages infected with WT L. monocytogenes for 180 minutes were used for Cytosolic Response determination). (B) Summary of the transcriptional responses of *IL1β*, *IFNβ*, and *IL6* in WT and *myd88* ^−/−^ macrophages during infection with either WT or *hly− L. monocytogenes*, at the indicated times post-infection (in minutes), determined by microarray analyses (Dataset S5). (C) Scatter plot representation of the transcriptional response of all genes defined as targets of the Vacuolar Response or the Cytosolic Response. Microarray data from *myd88* ^−/−^ macrophages infected with WT L. monocytogenes (Cytosolic Response-stimulating conditions) is plotted on the Y-axis, and microarray data from WT macrophages infected with *hly− L. monocytogenes* (Vacuolar Response-stimulating conditions) is plotted on the X-axis. Targets of only the Vacuolar Response are depicted as red squares (*e.g. IL1β*), targets of only the Cytosolic Response as blue triangles (*e.g. IFNβ*), and targets of both the Vacuolar Response and Cytosolic Response as purple diamonds (*e.g. IL6*).

We first determined the global transcriptional response of WT and *myd88* ^−/−^ macrophages infected with WT bacteria and *hly− L. monocytogenes*, using high-density oligonucleotide microarrays [33]. To identify genes induced by vacuolar bacteria, the transcriptional response of WT macrophages after 180 minutes of infection with *hly− L. monocytogenes* was subject to Significance Analysis of Microarrays (SAM) [34] (see Materials and Methods), and the resulting genes further selected to identify those with at least a 4-fold change in abundance. Using this approach, we identified 252 macrophage genes that met these criteria, which we defined as the “Vacuolar Response” of macrophages to L. monocytogenes (Figure 1A, “Vacuolar Response”; for full target gene list, see Dataset S1). This class of genes included many pro-inflammatory cytokines and chemokines, such as Interleukins 1α (*IL1α*) and 1β (*IL1β*), tumor necrosis factor (*TNF*), *KC*, and *MIP2*. Strikingly, none of the 252 genes of the Vacuolar Response were significantly induced in *myd88* ^−/−^ macrophages (Figure S1). *IL1β* is hereafter used as a representative target gene of the Vacuolar Response (Figure 1B, top panel).

To identify genes induced by cytosolic bacteria, the transcriptional response of *myd88* ^−/−^ macrophages after 180 minutes of infection with WT L. monocytogenes was subject to SAM, and the resulting genes further selected to identify those with at least a 4-fold change in abundance. Using this approach, we identified 106 macrophage genes, which we defined as the “Cytosolic Response” of macrophages to L. monocytogenes (Figure 1A, “Cytosolic Response”; for full target gene list, see Dataset S2). These genes were strongly induced starting at 2 hours post-infection, by which point WT bacteria were replicating robustly in the cytosol of infected cells. Among the genes most highly induced by the Cytosolic Response were Type I interferons, including *IFNβ* and multiple *IFNα* genes, and many known interferon-regulated genes. *IFNβ* is hereafter used as a representative target gene of the Cytosolic Response (Figure 1B, middle panel).

For 27 genes we observed induction by both the Vacuolar Response and the Cytosolic Response, as these genes were induced both in WT macrophages infected with *hly− L. monocytogenes* and in *myd88* ^−/−^ macrophages infected with WT L. monocytogenes. *IL6* is hereafter used as a representative target gene of this class (Figure 1B, bottom panel).

### Identification of the Primary Cytosolic Response

**Figure 2:**
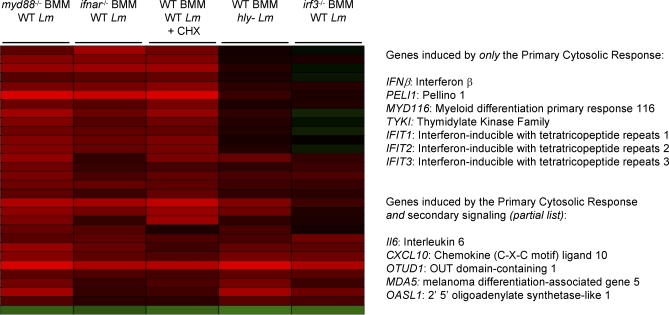
Determination of the Primary Cytosolic Response Cluster analysis of the microarray data used to determine the targets of the Primary Cytosolic Response, which are directly induced by NLR signaling in response to L. monocytogenes. The analyses used to determine these targets are described in Results. For the full target list, see Dataset S3.

Cytosolic Response targets included Type I interferons and Interleukin 6 (*IL6*), both of which induce the transcription of many additional genes in neighboring cells [15,35,36]. Five additional analyses were performed to identify genes directly induced by the host response to cytosolic bacteria, and not by secondary signaling. First, we required significant induction in infected *ifnar* ^−/−^ macrophages, which cannot respond to secreted Type I interferons. Second, induction in infected *ifnar* ^−/−^ macrophages must not have been significantly less than that in infected WT macrophages. These two filters removed genes significantly induced as a result of secondary Type I interferon signaling. Third, we required significant induction in infected WT macrophages treated with the protein synthesis inhibitor cycloheximide. Fourth, induction in cycloheximide-treated infected WT macrophages must not have been significantly less than induction in untreated infected WT macrophages. These two filters removed genes significantly induced as a result of secondary signaling by any other translated and secreted cytokine. Finally, we required that genes not have also been targets of the Vacuolar Response, as these genes would be induced in uninfected cells responding to extracellular TLR ligands. Ultimately, we identified only seven genes that met these five strict criteria, which we defined as targets of the Primary Cytosolic Response. These genes included *IFNβ*, *PELI1*, *MYD116*, *TYKI*, and three members of the *IFIT* (interferon-inducible with tetratricopeptide repeats) family (Figure 2). None of these genes were significantly induced in *irf3* ^−/−^ macrophages (data not shown). To identify genes induced by the Primary Cytosolic Response, but also induced by either secondary signaling or the Vacuolar Response, only criteria 1 and 3 (above) were used. By these relaxed criteria 20 additional genes were identified, including *IL6*, *CXCL10*, *OTUD1*, *MDA5*, *IGTP*, and *OASL1* (for full target gene list, see Dataset S3).

### *IRF3* Determines the Specificity of the Cytosolic Response

**Figure 3:**
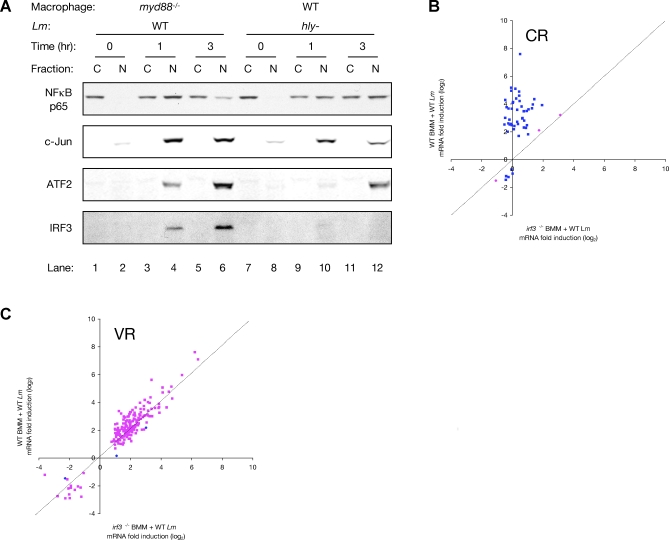
Specificity of the Cytosolic and Vacuolar Responses (A) Western blot analysis of the distribution of transcription factors NFκB p65, phospho-c-Jun, phospho-ATF2, and IRF3 in fractionated lysates of *myd88* ^−/−^ or WT macrophages infected with WT or *hly− L. monocytogenes*, respectively, for the indicated times (in hours). “C” indicates cytosolic fractions and “N” indicates nuclear fractions. Data is from the pooled lysates of two independent dishes. (B) Scatter plot representation of the transcriptional response of WT and *irf3* ^−/−^ macrophages infected for 3 hours with WT L. monocytogenes, as determined by microarrays. Shown are the responses of Cytosolic Response-specific target genes. Y-axis values are log_2_ fold change in RNA abundance in WT macrophages infected with WT L. monocytogenes. X-axis values are log_2_ fold change in RNA abundance in *irf3* ^−/−^ macrophages infected with WT L. monocytogenes. The superimposed dashed line has a slope = 1. Spots in blue are those identified by SAM as being significantly differently induced in the two conditions, while spots in pink are not different. (C) Scatter plot representation of the transcriptional response of WT and *irf3* ^−/−^ macrophages infected for 3 hours with WT L. monocytogenes, as determined by microarrays. Shown are the responses of Vacuolar Response-specific target genes. Y-axis values are log_2_ fold change in RNA abundance in WT macrophages infected with WT L. monocytogenes. X-axis values are log_2_ fold change in RNA abundance in *irf3* ^−/−^ macrophages infected with WT L. monocytogenes. The superimposed dashed line has a slope = 1. Spots in blue are those identified by SAM as being significantly differently induced in the two conditions, while spots in pink are not different.

The minimal overlap between the targets of the Vacuolar Response and the Cytosolic Response indicated a high degree of target gene specificity in these responses (Figure 1C). Stimulation of the Cytosolic Response during infection of *myd88* ^−/−^ macrophages with WT L. monocytogenes resulted in the activation and nuclear translocation of both NFκB and IRF3 (Figure 3A, lanes 1–6). The p65 subunit of NFκB was initially restricted to the cytosol of uninfected cells (lanes 1 and 2), but it accumulated in the nucleus as early as 1 hour post-infection (lanes 3 and 4), where it was still localized 3 hours post-infection (lanes 5 and 6). Similarly, IRF3 was observed in the nucleus of infected cells after 1 hour of infection (lanes 3 and 4), and by 3 hours accumulation of IRF3 in the nucleus had sharply increased (lanes 5 and 6). The transcription factors c-Jun and ATF2, both of which bind to the *IFNβ* promoter [16], also localized to the nucleus of infected cells during the Cytosolic Response (lanes 4 and 6). Stimulation of the Vacuolar Response during infection of WT macrophages with *hly− L. monocytogenes* (lanes 7–12) did not lead to nuclear localization of IRF3, which is consistent with failure of the Vacuolar Response to induce *IFNβ*. NFκB, c-Jun, and ATF2, however, were localized to the nucleus during the Vacuolar Response (lanes 10 and 12).

The induction of *IFNβ* during infection of macrophages with WT L. monocytogenes requires *IRF3* [24,37]. We further found that the majority of the Cytosolic Response is *IRF3*-dependent, as 94% of Cytosolic Response-specific target genes (*i.e.* genes that are not also targets of the Vacuolar Response, such as *IL6*) were significantly less induced in infected *irf3* ^−/−^ macrophages compared to infection of WT macrophages (Figure 3B).

WT bacteria in the cytosol of *myd88* ^−/−^ macrophages failed to induce the vast majority of Vacuolar Response genes, despite the robust nuclear localization of NFκB in these infected cells. The Cytosolic Response did not lead to feedback inhibition of Vacuolar Response target gene induction, unlike what has been reported during similar innate immune signaling in *Drosophila* [38]. Only 1.7% of Vacuolar Response-specific target genes showed greater induction during infection of *irf3* ^−/−^ macrophages, in which the Cytosolic Response was almost entirely absent, compared to infected WT macrophages (Figure 3C).

### Induction of the Cytosolic Response by Cytosolic Delivery of L. monocytogenes Genomic DNA Is Amplified by Co-Delivery of MDP

**Figure 4:**
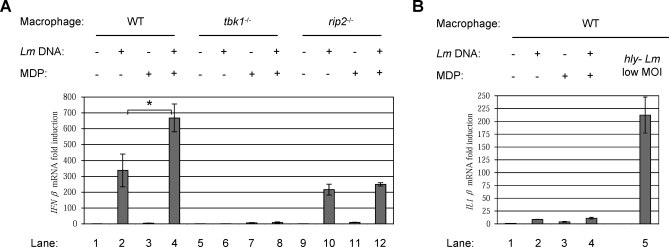
Cytosolic Delivery of L. monocytogenes DNA and Synthetic MDP Synergistically Induce the Cytosolic Response (A) Analysis by qPCR of *IFNβ* transcriptional induction in WT, *tbk1* ^−/−^, and *rip2* ^−/−^ macrophages 6 hours after transfection with the indicated combinations of L. monocytogenes genomic DNA (250 ng/ml) and synthetic MDP (10 μg/ml). The “*” indicates that these values are significantly different statistically with a p-value of 0.07. (B) Analysis by qPCR of *IL1β* transcriptional induction in WT macrophages 6 hours after transfection with the indicated combinations of L. monocytogenes genomic DNA and synthetic MDP, or 4 hours after infection with *hly− L. monocytogenes* at a low MOI.

To examine and dissect the Cytosolic Response, we attempted to recapitulate the host response to cytosolic bacteria by instead treating macrophages with purified bacterial ligands, using L. monocytogenes genomic DNA and synthetic MDP. When delivered directly into the cytosol of WT macrophages by transfection, L. monocytogenes DNA strongly induced the expression of *IFNβ* (Figure 4A, lane 2). However, co-transfection of macrophages with both L. monocytogenes genomic DNA and synthetic MDP yielded double the induction of *IFNβ* than that observed with DNA alone (lane 4), even though MDP alone yielded minimal *IFNβ* induction (lane 3). *IFNβ* induction was absolutely dependent on *TBK1* (lanes 6 and 8), a kinase required by all identified nucleic acid recognition receptors to activate the IRF3 transcription factor [37,39]. This defect was specific to the Cytosolic Response, as induction of Vacuolar Response target genes in response to *hly− L. monocytogenes* was unaffected in *tbk1^−/−^* cells (data not shown). Macrophages deficient for *RIP2*, an adaptor molecule required for *NOD2*-dependent NFκB activation [40,41], still induced *IFNβ* in response to DNA (lane 10), but co-transfection with MDP no longer produced any additional synergistic induction (lane 12). This response was independent of both *MyD88* and *TRIF* (Figure S2A), and was not due to contamination of the L. monocytogenes genomic DNA with other bacterial ligands, as we observed identical results with synthetic poly(dAT-dTA) DNA (Figure S2B). While virtually all Cytosolic Response-specific target genes were induced by transfection of macrophages with L. monocytogenes DNA, 94% of these genes were induced to an even greater magnitude by co-transfection of macrophages with both DNA and MDP (Figure S2C). Cytosolic delivery of DNA and MDP specifically induced Cytosolic Response genes without inducing Vacuolar Response genes, as these ligands only weakly induced *IL1β* (Figure 4B, compare lanes 2–4 to lane 5; Dataset S4).

### Nuclear NFκB Abundance Controls Synergistic Induction of the Cytosolic Response

**Figure 5:**
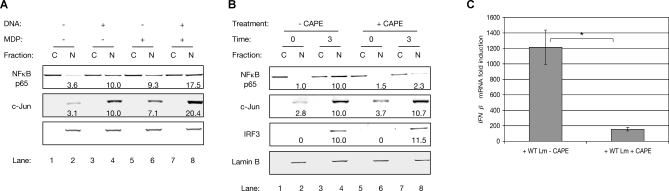
Induction of the Cytosolic Response Requires Nuclear NFκB (A) Quantitative Western blot analysis of NFκB p65, c-Jun, and ATF2 distribution in fractionated lysates of WT macrophages transfected with the indicated combinations of L. monocytogenes genomic DNA and synthetic MDP. “C” indicates cytosolic fractions and “N” indicates nuclear fractions. Nuclear abundance of proteins (indicated below the nuclear fraction within each blot) was normalized to the nuclear protein Lamin B, which also serves as fractionation control. For each transcription factor, abundance in the nucleus of macrophages transfected with L. monocytogenes DNA was arbitrarily set to 10.0, and all other abundances were calculated relative to this. Data was collected from two blots, with each blot using pooled lysates of two independent dishes. (B) Quantitative Western blot analysis of NFκB p65, c-Jun, and IRF3 protein distribution in fractionated lysates of WT macrophages infected with WT L. monocytogenes for the indicated times (in hours). Where indicated, cells were additionally treated with 10 μg/ml CAPE. (C) Analysis by qPCR of *IFNβ* transcriptional induction in WT macrophages infected with WT L. monocytogenes for 3 hours. Where indicated, cells were additionally treated with 10 μg/ml CAPE. The “*” indicates that these values are significantly different statistically with a p-value of 0.03.

Western blots were used to assess the activation of Cytosolic Response transcription factors by delivery of DNA and MDP. Transfection of WT macrophages with DNA alone (Figure 5A, lane 4) or MDP alone (lane 6) strongly activated NFκB to similar levels. When macrophages were transfected with both DNA and MDP, NFκB accumulated in the nucleus to a magnitude equal to the sum of that observed for the two ligands individually (lane 8). Similar results were observed for nuclear translocation of c-Jun (lanes 4, 6, and 8). No additive nuclear translocation of ATF2 was observed by co-delivery of both ligands (data not shown). MDP has never been found to activate IRF3 [8,24,28], consistent with our finding that MDP delivery alone does not induce *IFNβ* (Figure 4A, lanes 3 and 7).

To investigate the role of NFκB abundance in the synergistic induction of *IFNβ*, WT macrophages infected with WT L. monocytogenes were treated with 10 μg/ml caffeic acid phenyl ester (CAPE), a pharmacological inhibitor of NFκB nuclear trafficking [42]. CAPE treatment of infected macrophages caused a greater than 4-fold reduction in nuclear NFκB p65 accumulation, but had no effect on either c-Jun or IRF3 nuclear localization (Figure 5B). Compared to untreated macrophages, induction of *IFNβ* by L. monocytogenes in CAPE-treated macrophages was reduced greater than 7-fold (Figure 5C). Using DNA microarrays, we observed that this inhibition of NFκB nuclear translocation affected the entire Cytosolic Response, as 99% of Cytosolic Response-specific target genes were significantly less induced in infected CAPE-treated macrophages, compared to infected macrophages not CAPE-treated (Figure S3).

**Figure 6:**
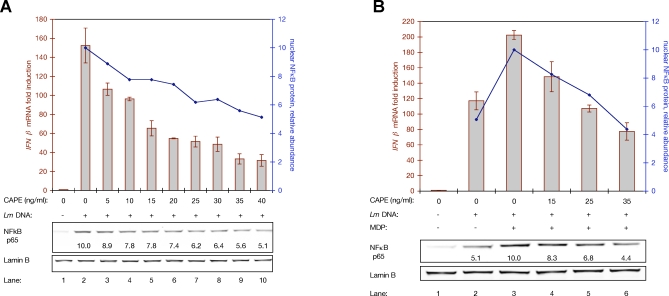
Synergistic Induction of *IFNβ* Is Sensitive to NFκB Nuclear Abundance (A) Dual analysis of *IFNβ* transcriptional induction and nuclear NFκB abundance in WT macrophages transfected with L. monocytogenes DNA, and additionally treated with CAPE at the indicated concentrations. *IFNβ* transcriptional analysis (red columns and left Y-axis) was by qPCR. Nuclear NFκB abundance (blue line and right Y-axis) was by quantitative Western blot analysis of the nuclear fractions of fractionated cells; blot is shown at the bottom, along with calculated relative NFκB abundances. (B) Dual analysis of *IFNβ* transcriptional induction and nuclear NFκB abundance in WT macrophages transfected with the indicated combinations of L. monocytogenes genomic DNA and MDP, and additionally treated with CAPE at the indicated concentrations. *IFNβ* transcriptional analysis (columns and left Y-axis, in red) was by qPCR. Nuclear NFκB abundance (line and right Y-axis, in blue) was by quantitative Western blot analysis; blot is shown at the bottom, along with calculated relative NFκB abundances.

Additionally, macrophages transfected with L. monocytogenes genomic DNA were treated with concentrations of CAPE ranging from 5 ng/ml to 40 ng/ml, which reduced nuclear NFκB abundance from 10%–50% compared to untreated cells (Figure 6A, lanes 3–10). These lower concentrations of CAPE allowed precise titration of nuclear NFκB, instead of the near complete inhibition observed previously with higher doses (Figure 5). Coincident with this reduction in nuclear NFκB abundance, induction of *IFNβ* decreased 30%–80%. At each increase in CAPE dosage, translocation of NFκB to the nucleus was reduced and induction of *IFNβ* declined. Similarly, macrophages transfected with either DNA alone or with both DNA and MDP were treated with a titration of CAPE. Without CAPE treatment, co-transfection of macrophages with both DNA and MDP yielded both twice the induction of *IFNβ* and twice the relocalization of NFκB to the nucleus, compared to transfection with DNA alone (Figure 6B, lanes 2 and 3). At increasing concentrations of CAPE, macrophages co-transfected with DNA and MDP induced less *IFNβ* (lanes 4–6). When macrophages co-transfected with DNA and MDP were treated with 25–35 ng/ml CAPE, the magnitude of nuclear NFκB matched that observed in untreated macrophages transfected only with DNA (compare lanes 5 and 6 to lane 2). Under these two conditions with equivalent NFκB activation—one with DNA alone and one with DNA, MDP, and CAPE—*IFNβ* induction was nearly identical.

### *NOD2* Is Required for Full Induction of *IFNβ* during Infection of Macrophages with Either L. monocytogenes or M. tuberculosis

To determine if L. monocytogenes release MDP during intracellular infection of macrophages, and if this is sensed by NOD2, *IFNβ* induction in *nod2* ^−/−^ macrophages infected with WT L. monocytogenes was assessed. Residual vacuolar signaling in these macrophages continued to activate NFκB, however, obscuring the contribution of *NOD2* signaling to nuclear NFκB abundance (Figure S4A). This residual TLR signaling was likely in response to the high concentrations of bacterial fragments delivered during the initial infection inoculum, and would be absent during a natural infection by a single bacterium. Therefore, to reduce NFκB activation by TLR overstimulation, macrophages were first tolerized by prior exposure to the synthetic TLR2 agonist Pam_3_CSK_4_ [43] (Figure S4B and S4C).

**Figure 7:**
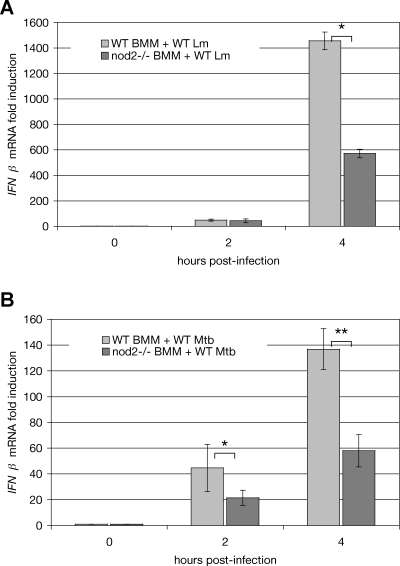
*NOD2* Is Required for Full *IFNβ* Induction during Infection with Either L. monocytogenes or M. tuberculosis (A) Analysis by qPCR of *IFNβ* transcriptional induction in tolerized WT and *nod2* ^−/−^ macrophages infected with WT L. monocytogenes. The “*” indicates that these values are significantly different statistically with a p-value of 0.003. (B) Analysis by qPCR of *IFNβ* transcriptional induction in tolerized WT and *nod2* ^−/−^ macrophages infected with WT M. tuberculosis. The “*” indicates that these values are significantly different statistically with a p-value of 0.05. The “**” indicates that these values are significantly different statistically with a p-value of 0.001.

In tolerized macrophages infected with WT L. monocytogenes, at 4 hours post-infection *nod2* ^−/−^ macrophages exhibited a greater than 2-fold reduction in *IFNβ* induction, as compared to WT macrophages (Figure 7A). Thus, *NOD2* signaling doubled the induction of *IFNβ* under these conditions. This exactly mirrored the effects of cytosolic co-delivery of DNA and MDP—a 2-fold amplification of *IFNβ* induction (Figure 4A) and a 2-fold amplification of NFκB nuclear abundance (Figure 5A), compared to the response elicited by bacterial DNA alone. To determine if *NOD2* signaling was necessary for the full induction of *IFNβ* in response to infection with other intracellular bacteria, we infected tolerized WT and *nod2* ^−/−^ macrophages with the pathogenic Gram-positive intracellular bacterium M. tuberculosis [44,45]. We observed that induction of *IFNβ* in *nod2* ^−/−^ macrophages infected with M. tuberculosis for 4 hours was less than half that observed in infected WT macrophages (Figure 7B).

## Discussion

### Identification of the Vacuolar and Cytosolic Responses to L. monocytogenes

In this study we have rigorously and comprehensively identified the Vacuolar and Cytosolic Responses of macrophages to infection with an intracellular bacterial pathogen. Our model system—primary bone marrow–derived macrophages of multiple genotypes infected with both WT L. monocytogenes and *hly−* bacteria—allowed precise separation of host responses to cytosolic and vacuolar bacteria. Previous genomic analyses of the macrophage transcriptional response to L. monocytogenes did not use *myd88* ^−/−^ macrophages to fully separate TLR and NLR signaling [23].

We have found that the Vacuolar Response was entirely *MyD88*-dependent, suggesting that the role of the TLR adaptor TRIF may be specific to innate immune responses to Gram-negative bacteria. The Vacuolar Response controlled the transcriptional induction of many pro-inflammatory cytokines, such as *IL1α*, *IL1β*, and *TNF*. In contrast, the *IRF3*-dependent Cytosolic Response induced a distinct and significantly non-overlapping set of 106 host response genes, including Type I interferons. We have further identified the 27 targets of the Cytosolic Response that were directly induced in response to cytosolic L. monocytogenes, and did not require secondary cytokine signaling for induction. Seven of these Primary Cytosolic Response target genes were induced entirely independent of any secondary signaling, and therefore were only induced directly in infected cells. Of these 7 genes, only *IFNβ* encodes a secreted protein, while many of the others encode potential regulators of signaling. For instance, *MYD116* is a homolog of *GADD34*, which can form a complex with protein phosphatase 1α to dephosphorylate eIF2α, thereby inhibiting protein synthesis [46]. *PELI1* is an E3 ubiquitin ligase that has been shown to modulate both TLR and IL1 signaling [47]. Consequently, the significant representation and robust induction of members of the *IFIT* family (*IFIT1–3*; induced 29.9-, 21.1-, and 13.9-fold, respectively) in this select group of genes warrants further study. Very little is known about IFIT proteins other than the potential of their tetratricopeptide repeats to mediate protein–protein interactions [48]. Despite their name, the *IFIT* genes responded directly to NLR signaling in response to L. monocytogenes.

We propose that these 7 targets of the Primary Cytosolic Response may provide a mechanism by which infected and uninfected cells could respond differently to the Type I interferons and other cytokines secreted during infection. Cytokines would trigger secondary signaling in all cells near the site of infection, but induction of *MYD116*, *PELI1*, *TYKI*, and *IFIT1–3* only in infected cells might modify this secondary signaling to trigger a different response. In this manner, infected cells could be specifically re-programmed to help contain infection.

We have found that two separable pathways coordinately control the Cytosolic Response. The first pathway consists of recognition of bacterial nucleic acid by a cytosolic pattern recognition receptor, perhaps DAI, that activates the transcription factors IRF3 and NFκB. We have demonstrated that L. monocytogenes genomic DNA induced Cytosolic Response target genes, and others have also shown that L. monocytogenes DNA induces *IFNβ* [12]. The response to both genomic DNA and live bacteria was *TBK1*-dependent (this study and [37]), and DAI has been shown to associate with TBK1 [14].

The second pathway consists of peptidoglycan fragment recognition by NOD2, which activates NFκB but not IRF3. We have demonstrated that the nuclear abundance of NFκB was limiting for the induction of *IFNβ*, and our data suggest that the two pathways converge by coordinate control of NFκB nuclear abundance. First, co-delivery of bacterial DNA and synthetic MDP doubled the nuclear abundance of NFκB, and induced twice as much *IFNβ*, compared to transfection of macrophages with DNA alone. Second, CAPE inhibited induction of *IFNβ*, and blocked only the nuclear translocation of NFκB. Third, under two conditions yielding equivalent NFκB activation—one with DNA alone and one with DNA, MDP, and CAPE—*IFNβ* induction was nearly identical, suggesting that control of NFκB abundance was the principal mechanism by which *NOD2* amplified *IFNβ* induction. The further activation of NFκB by *NOD2* signaling explains how this pathway contributed to *IFNβ* induction without activating IRF3.

We further found that *nod2* ^−/−^ macrophages, when TLR signaling was eliminated, induced significantly less *IFNβ* during infection with either L. monocytogenes or M. tuberculosis. We speculate that *NOD2* may play a similar role in response to other *IFNβ*-inducing intracellular bacteria whose peptidoglycan contains MDP, such as Francisella tularensis [36]. The convergence of NLR signaling pathways at the level of transcription factor abundance might allow complex signal integration in cells using a limited number of PRRs.

### Specificity in Target Gene Induction by the Vacuolar and Cytosolic Responses

The Vacuolar Response and Cytosolic Response controlled the transcription of largely distinct sets of target genes. The Vacuolar Response activated NFκB, and thereby induced pro-inflammatory cytokines such as *IL1β*, but did not activate IRF3, and hence did not activate *IRF3*-dependent targets including *IFNβ*. The Vacuolar Response was completely *MyD88*-dependent, and it has been demonstrated that a TLR-dependent but NFκB-independent remodeling of nucleosomes at the promoters of certain pro-inflammatory cytokines is required for induction [49–51]. Chromatin modifications are increasingly viewed as critical modulators ensuring appropriate control of inflammation [43], and may be a mechanism for determining target specificity of the Vacuolar and Cytosolic Responses. This may explain why activation of both NFκB and IRF3 during the Cytosolic Response did not induce *IL1β*, even though *IFNβ* was induced. Infection of *myd88* ^−/−^ macrophages with WT bacteria and the cytosolic delivery of purified ligands both bypassed TLR signaling, and therefore may not have triggered this nucleosome remodeling.

The OspF virulence factor injected by the bacterial pathogen Shigella flexneri modifies host chromatin during infection to block the activation of certain NFκB target genes [52], but we have no evidence that L. monocytogenes possesses analogous effector molecules. Instead, by escaping the vacuole, L. monocytogenes may avoid induction of inflammation by taking advantage of the inherent Cytosolic Response target specificity. Previous studies in which MDP was observed to induce pro-inflammatory cytokines used non-primary, non-immune system cells, and often assessed host response as late as 24 hours after MDP delivery, all of which could have resulted in significantly altered chromatin states [8,9,53–55].

### The Role of *NOD2* in Bacterial Pathogenesis

The role of *NOD2* in innate immunity is controversial [56,57]. A previous study found that *NOD2* was involved in the mouse innate immune response to L. monocytogenes, but only during intragastric infection [32]. This *in vivo* infection model may be particularly important for understanding the pathogenesis of food-borne bacteria such as L. monocytogenes. In certain cells of the gut epithelium, through which L. monocytogenes must pass in an oral model of infection, expression of many TLRs is naturally downregulated, possibly to reduce inappropriate responses to commensal flora [58]. This is consistent with our finding that *NOD2*-mediated synergistic induction of *IFNβ* in response to intracellular bacterial infection was only manifest when TLR signaling was pharmacologically suppressed, and may also explain the results of a previous study in which *rip2* ^−/−^ macrophages with intact TLR signaling had no defect in their response to L. monocytogenes [37]. Curiously, while *irf3* ^−/−^ and *ifnar* ^−/−^ mice infected intravenously with L. monocytogenes were more resistant to infection [59–61], *nod2* ^−/−^ mice infected intragastrically were more susceptible [32]. Given our results showing that infected *nod2* ^−/−^ macrophages induced less *IFNβ* than infected WT macrophages, the role of Type I interferons in intracellular bacterial pathogenesis may differ depending on the *in vivo* model used.

## Materials and Methods

For more detailed versions of many of the following methods, please see: http://microbiology.berkeley.edu/ppmicroarrayhybridprotoc.htm.

### Cell culture and bacteria.

Macrophages were derived from the bone marrow of mice over 7 days in media composed of DMEM, 2 mM glutamine, 1 mM pyruvate, 10% CSF from 3T3 cells, 20% heat inactivated FBS, and penicillin-streptomycin. For all experiments, macrophages were grown in identical media without penicillin-streptomycin. For infections, WT 10403S (DP-L184) and *hly−* (DP-L2161) L. monocytogenes were grown to mid-log in BHI media at 30 °C with shaking. Bacteria were then PBS-washed, resuspended in PBS at a normalized OD_600_ = 1.2, and added to macrophages at a 1:50 volume:volume ratio, resulting in > 75% of macrophages infected with at least one bacterium. For experiments with “low MOI” infection, and experiments involving Pam_3_CSK_4_-pre-treated macrophages, resuspended bacteria were added at a 1:1000 ratio. At 30 minutes post-infection macrophages were washed 3 times with fresh pre-warmed media, and at 60 minutes post-infection gentamicin was added to a final concentration of 50 μg/ml. For experiments containing a 30 minute post-infection time point, macrophages were instead washed at 20 minutes and gentamicin added at 30 minutes. CAPE (Calbiochem) (or EtOH for mock-treated controls) was added 60 minutes post-infection. For tolerization of cells, Pam_3_CSK_4_ (Invivogen) was added at a final concentration of 100 ng/ml, 24 hours prior to the subsequent experiment. Where indicated, cycloheximide (or water for mock-treated controls) was added at a final concentration of 10 μg/ml 30 minutes before infection with bacteria, and added back after the washes at t = 30 minutes. This treatment reduces macrophage translation by 94.5% (determined by ^35^S-Met incorporation, data not shown).

For infections with M. tuberculosis, macrophages were infected with the Erdman strain as previously described [45]. Briefly, M. tuberculosis cultures were washed with PBS and resuspended in DMEM supplemented with 10% horse serum. Pam_3_CSK_4_-pre-treated macrophages were incubated with bacteria in DMEM + 10% horse serum for 2 hours at an MOI of 10. Cells were then washed three times with PBS and returned to macrophage media.

### Mice.

All macrophages were from mice in the C57BL/6 genetic background, including femurs from knockout mice (see Acknowledgments).

### Macrophage RNA preparation.

Macrophage RNA was isolated with the Ambion RNAqueous kit (Applied Biosystems) according to the manufacturer's protocol, after first treating the cells with Ambion RNAlater (Applied Biosystems). For microarray experiments, RNA was amplified to generate amplified RNA (aRNA) using the Ambion Amino-Allyl Message Amp II aRNA Amplification Kit (Applied Biosystems) according to the manufacturer's protocol. For qPCR experiments, RNA was DNase treated with the Ambion TURBO DNA-free kit (Applied Biosystems).

### Microarrays.

Microarrays were printed at the UCSF Center for Advanced Technology, using the MEEBO 70-mer oligonucleotide set (Illumina; for more details see http://alizadehlab.stanford.edu/). Microarray probes were generated by coupling aRNA to Cy dye mono-functional NHS esters (Amersham) using 100 mM sodium bicarbonate (pH 9.0) and 50% DMSO. 5 μg of Cy5-coupled sample and 5 μg Cy3-coupled reference (generated by pooling an equal volume of every sample used in a given set of arrays) were hybridized to MEEBO microarrays at 63 °C for 2 days. After washing, arrays were scanned on a GenePix 4000B scanner (Molecular Devices). Arrays were gridded with SpotReader software (Niles Scientific). Acquisition of data was performed using the GenePix Pro 6 software package (Molecular Devices). Data was normalized by first using stringent criteria to identify a subset of features of highest quality, and then calculating a normalization factor such that the ratio of medians of the Cy5 and Cy3 values for these features = 1. This ratio was then applied to all the features. Features not meeting minimum criteria to assure quality were removed from the datasets. These criteria are available upon request. Hierarchical Pearson clustering and other analyses were performed with the Acuity 4 software package (Molecular Devices). The determination of Vacuolar Response and Cytosolic Response target genes was each from multiple arrays representing four independent experiments (*e.g.* 4 independent dishes of uninfected *myd88* ^−/−^ macrophages and 4 independent dishes of *myd88* ^−/−^ macrophages infected with WT L. monocytogenes for 180 minutes were used for Cytosolic Response determination), and other determinations were from 2–4 independent experiments. Fold change in RNA abundance is relative to uninfected samples. SAM analysis (Stanford University) [34] was performed with two-class unpaired designs to identify genes that were differently expressed in infected versus uninfected macrophages. For initial target gene discovery (defining the Vacuolar Response and Cytosolic Response), the false discovery rate (FDR) was set to 1%. For subsequent SAM analyses the FDR was set to 10%. Information linked to each unique Oligo ID can be found at http://meebo.ucsf.edu:8080/meebo/meeboInfo.jsp?oligoid=(insert Oligo ID here). The GEO accession number for the primary array data is GSE8104. Accession numbers for genes of the Primary Cytosolic Response are as follows: NM_010510 (*IFNβ*), NM_008331 (*IFIT1*), NM_008332 (*IFIT2*), NM_010501 (*IFIT3*), NM_020557 (*TYKI*), NM_030015 (*PELI1*), NM_008654 (*MYD116*), NM_031168 (*IL6*), BC030067 (*CXCL10*), XM_130015 (*OTUD1*), NM_027835 (*MDA5*), NM_018738 (*IGTP*), and NM_145209 (*OASL1*). See Dataset S3 for more information on the Primary Cytosolic Response target genes.

### qPCR.

DNase-treated macrophage RNA was reverse transcribed and subject to quantitative PCR using DyNAmo SYBR Green 2-step qRT-PCR reagents (NEB/Finnzyme) and was performed on an Mx3000P machine and analyzed using MxPro software (Stratagene). The sequences of gene-specific primers are as follows: tggcattgttaccaactgggacg (5' *β-actin*), gcttctctttgatgtcacgcacg (3' *β-actin*), gcactgggtggaatgagactattg (5' *IFNβ*), ttctgaggcatcaactgacaggtc (3' *IFNβ*), gacctgttctttgaagttgacgg (5' *IL1β*), and tgtcgttgcttggttctccttg (3' *IL1β*). All RNA abundances are normalized to *β-actin*, and fold induction is relative to mock-infected samples. Data shown is the mean of at least three, and in most cases four, independent experiments (except for the data shown in Figure 5C, which represents the average of two independent experiments), and error bars represent the standard error of the mean. Statistical analyses (t-Tests with equal variances assumed) were performed using the Analysis Package Add-In feature of Microsoft Excel, and p-values are noted in figure legends.

### Cell fractionation and Western blots.

Macrophages were fractionated using NE-PER reagents (Pierce) supplemented with HALT protease inhibitors (Pierce) following the manufacturer's protocol. Protein concentration was determined using BCA reagents (Pierce) and equal masses of protein were run on 10% NuPAGE bis-tris gels (Invitrogen). Blots were probed with the following primary antibodies, all from Santa Cruz Biotech: anti-NFκB p65 (sc-372X), anti-IRF3 (sc-9082X), anti phospho-c-Jun (sc-16312X), anti-phospho-ATF2 (sc-8398X), and anti-Lamin B (sc-6217). Protein blots were probed with a secondary antibody covalently attached to an infrared emitting fluorphor (IRdye-680 and IRdye-800, Li-Cor Biosciences). Blots were scanned using the Odyssey Infrared Imaging System (Li-Cor Biosciences), and quantitated using the accompanying software package. Relative protein abundances were normalized to Lamin B. Data was collected from two blots, with each blot using pooled lysates of two independent dishes (four dishes total, except for the data shown in Figure 2A, which is from two independent dishes). The non-quantitative Western blot shown in Figure 2A was instead probed with an HRP-coupled secondary antibody, and developed with the ECL Plus Western Blot Detection System (GE Healthcare).

### Transfections.

Macrophages were transfected using FuGene 6 Transfection Reagent (Roche) according to the manufacturer's protocol. L. monocytogenes genomic DNA was prepared by manual disruption of mid-log WT 10403S bacteria using glass beads and phenol:chloroform. DNA was extracted 3 times with phenol:chloroform, treated with RNases A and H, and extracted again with phenol:chloroform. DNA was then digested to completion using EcoRI and BamHI, yielding fragments averaging 1–5 kb, extracted with phenol:chloroform, and precipitated. All phenol:chloroform extractions were done using PhaseLock gel (Eppendorf). DNA was dissolved in pyrogen-free water and used at a final concentration of 250 ng/ml. MDP (Calbiochem) was used at final concentration of 100 μg/ml. Poly(dAT-dTA) double-stranded nucleic acid (Amersham) was dissolved in 100 mM NaCl and used at a final concentration of 250 ng/ml. All transfection experiments were done for 6 hours. CAPE (Calbiochem) (or EtOH for mock-treated controls) was added at the indicated concentration 180 minutes post-infection. Cycloheximide (or water for mock-treated controls) was added at a final concentration of 10 μg/ml 180 minutes after initial ligand transfection.

## Supporting Information

Dataset S1 (34 KB XLS)

Dataset S2 (18 KB XLS)

Dataset S3 (20 KB XLS)

Dataset S4 (48 KB XLS)

Dataset S5 (124 KB XLS)

Figure S1 The Vacuolar Response Is Completely *MyD88*-Dependent Scatter plot representation of microarray data from *myd88* ^−/−^ macrophages infected with *hly− L. monocytogenes* (see Dataset S5). Shown are the responses of Vacuolar Response-specific target genes. Y-axis values are log_2_ fold change in RNA abundance in WT macrophages infected with *hly− L. monocytogenes*. X-axis values are log_2_ fold change in RNA abundance in *myd88* ^−/−^ macrophages infected with *hly− L. monocytogenes*. Spots in blue are those identified by SAM as being significantly differently induced in the two conditions (all spots).
(40 KB PDF)

Figure S2 *MyD88*- and *TRIF*-Independent Synergistic Induction of the Entire Cytosolic Response by L. monocytogenes Genomic DNA and Synthetic MDP (A) Analysis by qPCR of *IFNβ* transcriptional induction in *myd88* ^−/−^ *trif* ^−/−^ macrophages transfected with the indicated combinations of L. monocytogenes genomic DNA and synthetic MDP.
(B) Analysis by qPCR of *IFNβ* transcriptional induction in *myd88* ^−/−^ *trif* ^−/−^ macrophages transfected with the indicated combinations of synthetic poly(dAT-dTA) double-stranded DNA and synthetic MDP.
(C) Scatter plot representation of the transcriptional response of WT macrophages transfected with either L. monocytogenes genomic DNA alone, or with both L. monocytogenes DNA and MDP, as determined by microarrays. Shown are the responses of Cytosolic Response-specific target genes. Y-axis values are log_2_ fold change in RNA abundance in WT macrophages transfected with L. monocytogenes DNA, and X-axis values are log_2_ fold change in RNA abundance in WT macrophages transfected with both L. monocytogenes DNA and synthetic MDP. Spots in blue are those identified by SAM as being significantly differently induced in the two conditions, while spots in pink are not different. The superimposed dashed line has a slope = 1. The superimposed solid black line represents the best fit of the observed transcriptional responses, and has a slope = 0.71 (see Dataset S4 for complete array data).
(56 KB PDF)

Figure S3 Induction of the Cytosolic Response Requires NFκB Scatter plot representation of the transcriptional response of WT macrophages infected for 3 hours with WT L. monocytogenes, and where indicated additionally treated with 10 μg/ml CAPE. Shown are the responses of Cytosolic Response-specific target genes. Transcription responses were determined by microarrays. Y-axis values are log_2_ fold change in RNA abundance in WT macrophages infected with WT L. monocytogenes. X-axis values are log_2_ fold change in RNA abundance in WT macrophages infected with WT L. monocytogenes and additionally treated with CAPE. The superimposed dashed line has a slope = 1. Spots in blue are those identified by SAM as being significantly differently induced in the two conditions, while spots in pink are not different.
(12 KB PDF)

Figure S4 Tolerization of Macrophages with Prior Exposure to Pam_3_CSK_4_
We previously observed that WT macrophages infected with WT L. monocytogenes exhibited persistent TLR-mediated signaling, even at later time points. From our microarray analyses, we found that L. monocytogenes induced *IL1β* exclusively by *MyD88*-dependent signaling pathways (summarized in Figure 1B). However, *IL1β* was still strongly induced at later time points in WT macrophages infected with WT L. monocytogenes, when the bacteria were in the cytosol. This suggested that these cells continued TLR signaling from the vacuole even after the bacteria had escaped from this compartment. As TLR signaling strongly activated NFκB (Figure 2A), we considered it likely that this residual TLR signaling masked any contribution by *NOD2* signaling to nuclear NFκB. Indeed, we found that both WT and *nod2* ^−/−^ macrophages infected with WT L. monocytogenes induced *IFNβ* to nearly identical levels (Figure S4A). To remove residual TLR signaling in these cells we treated macrophages with Pam_3_CSK_4_, a synthetic TLR2 agonist. Pretreatment for 24 hours with Pam_3_CSK_4_ had previously been shown to “tolerize” macrophages, causing their TLR signaling to dampen after transitory stimulation [43]. Compared to non-tolerized macrophages, Pam_3_CSK_4_-treated WT macrophages almost fully ceased the induction of *IL1β* 2 hours after infection with WT L. monocytogenes (Figure S4B). These tolerized macrophages were still capable of appropriate TLR-mediated signaling, as we observed robust and sustained induction of *IL1β* during infection of Pam_3_CSK_4_-treated WT macrophages with *hly− L. monocytogenes*, which remain in the vacuole (Figure S4C).
(A) Analysis by qPCR of *IFNβ* transcriptional induction in non-tolerized WT and *nod2* ^−/−^ macrophages infected with WT L. monocytogenes.
(B) Analysis by qPCR of *IL1β* transcriptional induction in WT macrophages infected with WT L. monocytogenes, and where indicated additionally tolerized 24 hours pre-infection with 100 ng/ml Pam_3_CSK_4_.
(C) Analysis by qPCR of *IL1β* transcriptional induction in WT macrophages tolerized 24 hours pre-infection with 100 ng/ml Pam_3_CSK_4_, and infected with either WT L. monocytogenes or *hly− L. monocytogenes*.
(43 KB PDF)
