# Supplementary material for: Distinct TLR- and NLR-Mediated Transcriptional Responses to an Intracellular Pathogen
Source: PLoS Pathog. 2008 Jan 11;4(1):e6. doi: 10.1371/journal.ppat.0040006 (PMC2186359; doi:10.1371/journal.ppat.0040006)
Supplement: Supplementary file 2 [file ppat.0040006.sg002.pdf]

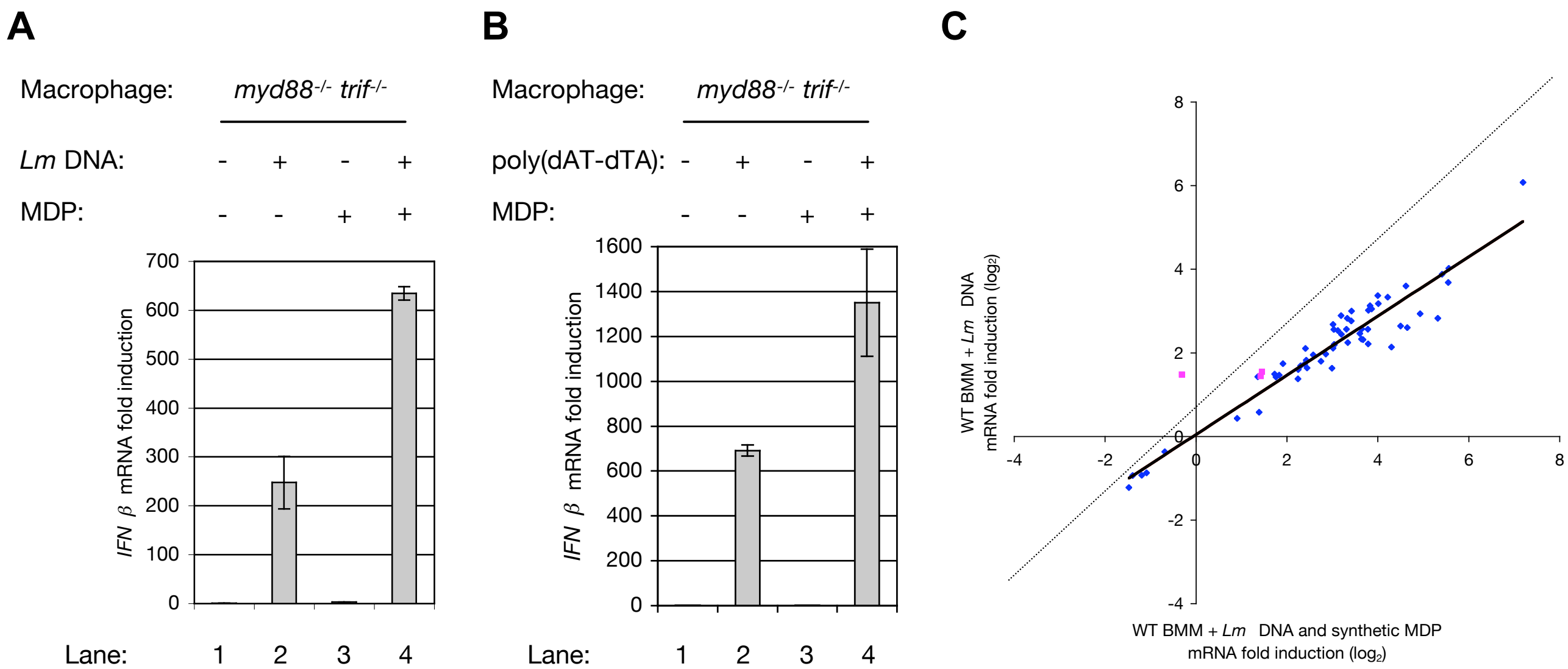
A
Macrophage: myd88-/- trif-/-
Lm DNA: - + - +
MDP: - - + +
IFN β mRNA fold induction
0
100
200
300
400
500
600
700
Lane: 1 2 3 4
B
Macrophage: myd88-/- trif-/-
poly(dAT-dTA): - + - +
MDP: - - + +
IFN β mRNA fold induction
0
200
400
600
800
1000
1200
1400
1600
Lane: 1 2 3 4
C
WT BMM + Lm DNA
mRNA fold induction (log2)
WT BMM + Lm DNA and synthetic MDP
mRNA fold induction (log2)
-4
-2
0
2
4
6
8
