# Supplementary material for: Distinct TLR- and NLR-Mediated Transcriptional Responses to an Intracellular Pathogen
Source: PLoS Pathog. 2008 Jan 11;4(1):e6. doi: 10.1371/journal.ppat.0040006 (PMC2186359; doi:10.1371/journal.ppat.0040006)
Supplement: Supplementary file 3 [file ppat.0040006.sg003.pdf]

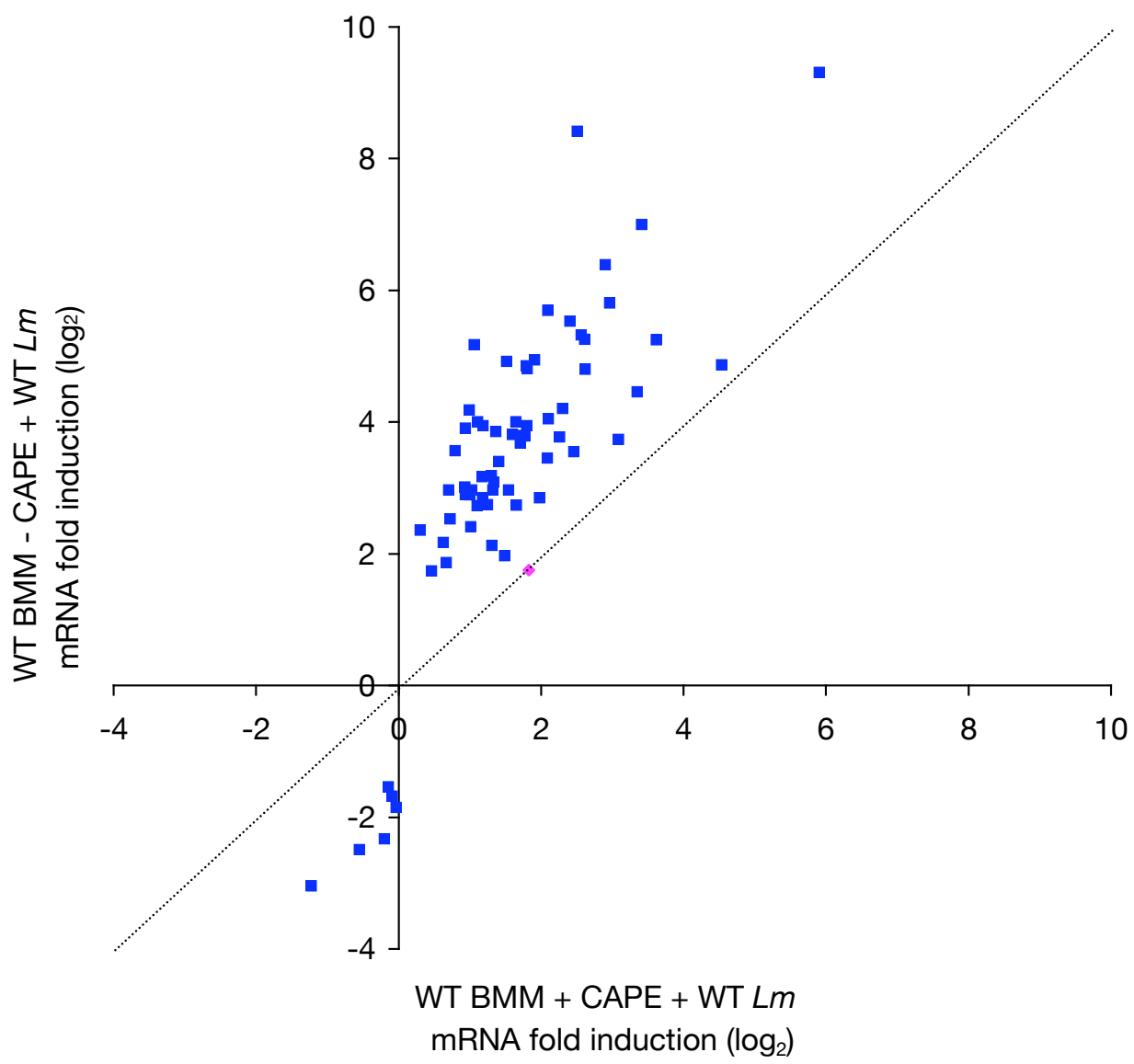

WT BMM - CAPE + WT *Lm*
mRNA fold induction (log_2)
WT BMM + CAPE + WT *Lm*
mRNA fold induction (log_2)
10
8
6
4
2
0
-2
-4
-4
-2
0
2
4
6
8
10
