# Supplementary material for: Distinct TLR- and NLR-Mediated Transcriptional Responses to an Intracellular Pathogen
Source: PLoS Pathog. 2008 Jan 11;4(1):e6. doi: 10.1371/journal.ppat.0040006 (PMC2186359; doi:10.1371/journal.ppat.0040006)
Supplement: Supplementary file 4 [file ppat.0040006.sg004.pdf]

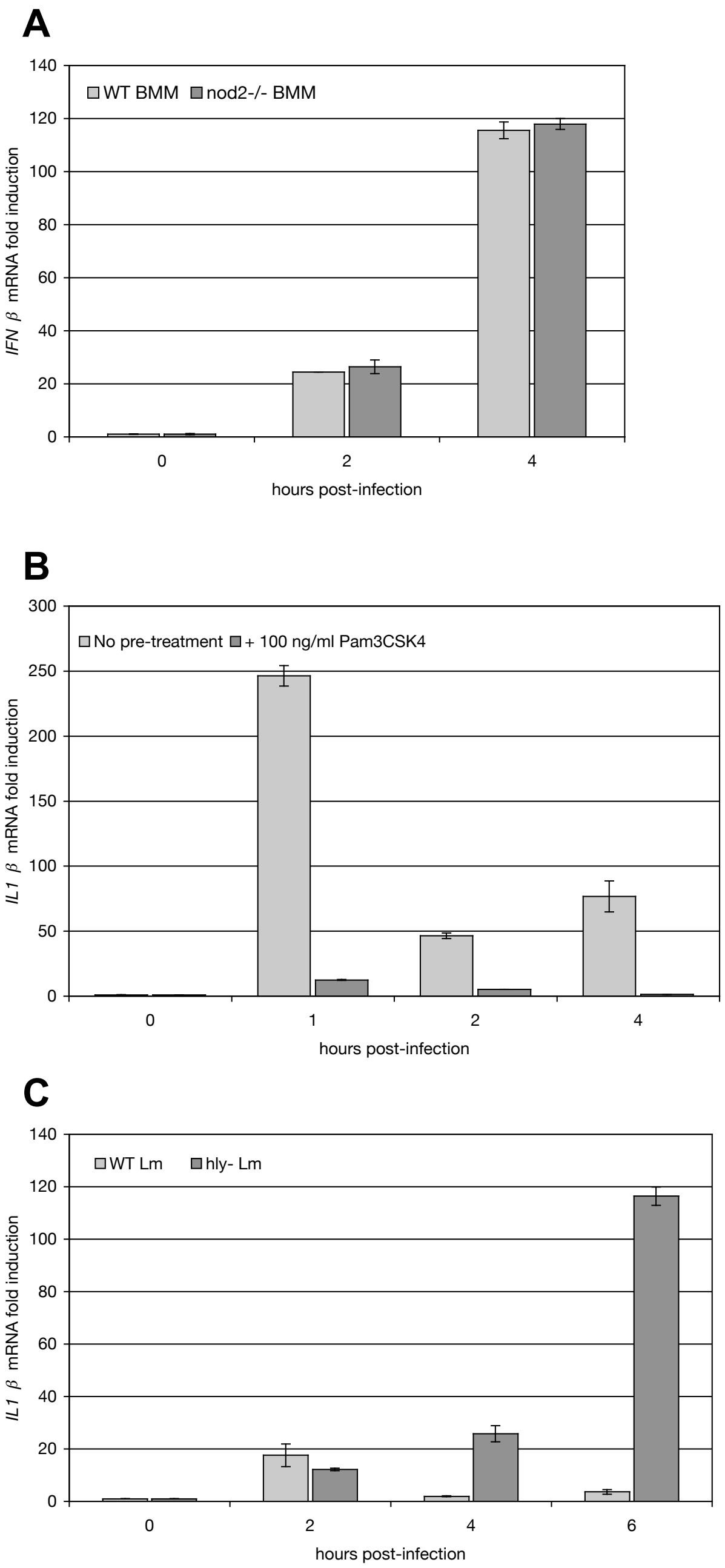
A
WT BMM
nod2-/- BMM
IFN β mRNA fold induction
0
20
40
60
80
100
120
140
0
2
4
hours post-infection
B
No pre-treatment
+ 100 ng/ml Pam3CSK4
IL1 β mRNA fold induction
0
50
100
150
200
250
300
0
1
2
4
hours post-infection
C
WT Lm
hly- Lm
IL1 β mRNA fold induction
0
20
40
60
80
100
120
140
0
2
4
6
hours post-infection
